# The neural basis of neuropsychiatric symptoms in Alzheimer’s disease

**DOI:** 10.3389/fnagi.2024.1487875

**Published:** 2024-12-05

**Authors:** Nicole K. Zhang, Selena K. Zhang, Li I. Zhang, Huizhong W. Tao, Guang-Wei Zhang

**Affiliations:** ^1^Zilkha Neurogenetic Institute, Keck School of Medicine, University of Southern California, Los Angeles, CA, United States; ^2^Biomedical Engineering Program, Viterbi School of Engineering, University of Southern California, Los Angeles, CA, United States; ^3^Department of Physiology and Neuroscience, Keck School of Medicine, University of Southern California, Los Angeles, CA, United States

**Keywords:** Alzheimer’s disease, neuropsychiatric symptoms, anxiety, depression, apathy, emotion, cortex, pathology

## Abstract

Neuropsychiatric symptoms (NPS) such as depression, anxiety, apathy and aggression affect up to 90% of Alzheimer’s disease (AD) patients. These symptoms significantly increase caregiver stress and institutionalization rates, and more importantly they are correlated with faster cognitive decline. However, the neuronal basis of NPS in AD remains largely unknown. Here, we review current understanding of NPS and related pathology in studies of AD patients and AD mouse models. Clinical studies indicate that NPS prevalence and severity vary across different AD stages and types. Neuroimaging and postmortem studies have suggested that pathological changes in the anterior cingulate cortex, hippocampus, prefrontal cortex, and amygdala are linked to NPS, although the precise mechanisms remain unclear. Studies of AD mouse models have indicated that amyloid-beta and tau-related neurodegeneration in the hippocampus, prefrontal cortex, and anterior cingulate cortex are correlated with NPS-like behavioral deficits. A better understanding of the NPS phenotypes and related pathological changes will pave the way for developing a better management strategy for NPS in AD patients.

## Introduction

Alzheimer’s disease (AD) is a neurodegenerative disease and the leading cause of dementia, contributing to around 70% of dementia cases (Gooch et al., 2017). Around 6.7 million Americans aged 65 and older have AD or AD-related dementia (No author list, 2023) and this number is expected to continue to grow (Brookmeyer et al., 2018). AD is primarily known for its progressive cognitive impairment, but it also has an underrecognized neuropsychiatric component that significantly impacts patients and caregivers, which contributes to the increased caregiver burden, higher institutionalization rates, and a diminished functional state (Wadsworth et al., 2012; Mendez, 2021; Chen et al., 2022). Neuropsychiatric symptoms in AD patients primarily include depression, anxiety, agitation, aggression, apathy, hallucination, and delusion (Chen et al., 2021; Ismail et al., 2022). NPS can be present in up to 90% of AD patients (Chen et al., 2021) and could be manifested at all stages of AD. NPS prevalence, severity, and timeline may differ depending on specific types of AD. For example, early-onset AD patients are more likely to develop depression than those with late-onset AD (Liu et al., 2023), suggesting shared or correlated pathological changes. Moreover, clinical studies have indicated that NPS, especially depression, are risk factors for cognitive decline and more rapid progression of the disease (Umucu et al., 2019; Mendez, 2021; Wallensten et al., 2023). Despite their prevalence and profound effects on the quality of life, NPS in AD receive considerably less research and clinical attention compared to the extensively characterized cognitive decline.

Although there have been extensive studies on the cellular and molecular mechanisms of AD, such as amyloid accumulation and neuroinflammation, few have been framed under the context of NPS (Krell-Roesch et al., 2019; Chen et al., 2021; Schaffer Aguzzoli et al., 2023). While there are human neuroimaging studies that investigate the NPS-associated pathological changes of candidate brain regions (Chen et al., 2021), the question of how exactly these changes contribute to the progression of AD and NPS remains yet unaddressed. Moreover, it is unclear how the pathological and molecular changes affect specific neural circuits that play a role in regulating these symptoms. Mouse models have provided unique opportunities to explore neural circuitry mechanisms underlying neuropsychiatric symptoms. Due to their sheer variety, they are invaluable tools for investigating the complex interactions between AD and NPS. Utilization of these models may have the potential to develop unique biomarkers and tailored interventions to alleviate the adverse effects of NPS.

In this narrative review, we focus on NPS in AD, summarizing current findings on NPS-related neural circuitry changes in AD patients and AD mouse models, while discussing the challenges associated with these studies. We highlight the difficulties in quantifying NPS-related behavioral deficits in animal models and the existence of inconsistencies in the behavioral phenotypes across different models. As such, the choice of behavioral tests poses challenges for advancing our understanding of the circuit mechanisms underlying AD (Geda et al., 2013; Zhao et al., 2016; Pless et al., 2023).

## Prevalence and progression of NPS in AD patients

**FIGURE 1:**
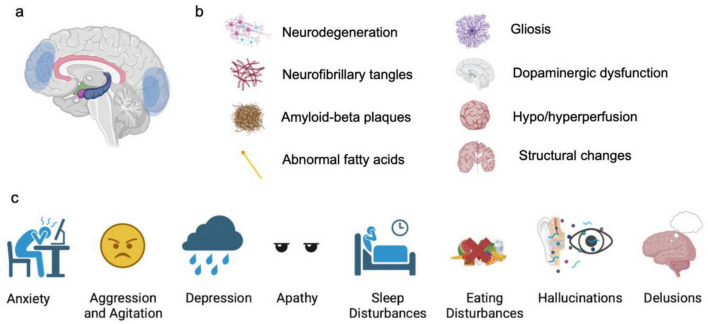
Typical NPS in AD patients. **(a)** Commonly affected brain regions, based on neuroimaging findings. **(b)** Pathological changes associated with NPS. **(c)** Types of NPS.

Apathy, depression, anxiety, and agitation/aggression manifest early in AD, while hallucination and delusion generally occur at a later stage, often worsening as the disease progresses. Understanding the prevalence and trajectory of these symptoms is critical for developing effective management and care strategies. NPS in AD patients are assessed with a variety of questionnaires and surveys, including the Neuropsychiatric Inventory and Cornell Scale for Depression in Dementia (Table 1 and Figure 1).

**TABLE 1:** NPS in AD patients.

NPS category	Specific description	Human metrics	Prevalence	Candidate brain structures	Neuroimaging findings	Pathological changes from postmortem studies	Corresponding citations
Apathy	Decreased initiative, less affection, social withdrawal, and lacking in emotions	Neuropsychiatric Index, Apathy Evaluation Scale, Hospital Anxiety and Depression Scale, Dementia Apathy Interview and Rating	19–88%	Anterior Cingulate Cortex, Prefrontal Regions, Putamen, Caudate Nucleus	Cortical thinning, atrophy, low FA scores, amyloid accumulation, hypometabolism, NFT burden	Dopaminergic dysfunction in basal ganglia, anterior cingulate, and frontal cortices, upregulated 5-HTT sites	Lyketsos et al., 2002; Marshall et al., 2006, 2007, 2013; Starkstein et al., 2006; Apostolova et al., 2007; Bruen et al., 2008; Wetzels et al., 2010; Kim et al., 2011; Mitchell et al., 2011; Tighe et al., 2012; Mori et al., 2014; Zhao et al., 2016; Huey et al., 2017; Le Heron et al., 2018; Yasuno et al., 2020; Chen et al., 2021; Grossman et al., 2021; Mintzer et al., 2021; Eikelboom et al., 2022; Johansson et al., 2022; Lanctôt et al., 2023; Custo et al., 2024
Depression	Diminished interest, weight changes, insomnia/hypersomnia, psychomotor agitation/retardation, and fatigue	Beck Depression Inventory, Hamilton Depression Rating Scale, Neuropsychiatric Inventory, Cornell Scale for Depression in Dementia, Geriatric Depression Scale	∼40%; 32% in MCI; 38–41% in AD stages	ACC, Prefrontal Cortex, Hippocampus, Striatum, Amygdala, Orbitofrontal Cortex	Hypometabolism, structural changes, gray matter atrophy, white matter lesions, altered functional connectivity	White matter hyperintensity in occipital area, chronic cerebral hypoperfusion, NFT burden, serotonergic dysfunction, abnormal fatty acids, reduction of antioxidant enzymes	Lee et al., 2012; Zhao et al., 2016; Ismail et al., 2017; Leung et al., 2021; Sáiz-Vazquez et al., 2021; Botto et al., 2022; Eikelboom et al., 2022; Lanctôt et al., 2023
Anxiety	Excessive uneasiness/tenseness, avoidance of specific situations, separation anxiety from caregivers	Neuropsychiatric Index, Rating Anxiety in Dementia, Hamilton Anxiety Rating Scale, Beck Anxiety Index, Behavior Pathology in AD Scale	26.1–70%	Hippocampus, Entorhinal Cortex, Amygdala, Prefrontal Cortex	Decreased volume, atrophy, hypometabolism	High amyloid-beta accumulation, NFT and AB in neocortex, limbic regions, anterior cingulate, entorhinal cortex, serotonergic transporters in the temporal cortex	Teri et al., 1999; Porter et al., 2003; Zhao et al., 2016; Cummings, 2020; Leung et al., 2021; Mendez, 2021; Botto et al., 2022; Várkonyi et al., 2022
Agitation and aggression	Excessive verbal/motor activity without focus; physical/verbal behavior intended to cause harm	Neuropsychiatric Inventory, Present Behavioral Examination, Overt Aggression Scale, Irritability Scale, Cohen Mansfield Agitation Inventory	7.4% in MCI; 70% in AD	Amygdala, Anterior Cingulum, Hippocampus, Frontal Insular, Frontal Cortex	Atrophy, low fractional anisotropy, increased functional connectivity, amyloid accumulation, hypometabolism	Lowered ChAT activity in frontal and temporal cortex, serotonergic dysfunction, 5-HTT sites preserved, 5-HIAA scores inversely correlated with agitation	Deutsch et al., 1991; Trzepacz et al., 2013; Ehrenberg et al., 2018; Choi et al., 2019; Yu et al., 2019; de Mauleon et al., 2020
Sleep disturbance	Typically increased amount of sleep and early awakenings; less commonly, night-time awakenings	Neuropsychiatric Index	24.5–39%	Right Middle Frontal Gyrus, Anterior Cingulate Cortex	Hyperperfusion, hypometabolism	Decreased melatonin in the CSF	Liu et al., 1999; McCurry and Ancoli-Israel, 2003; Ismail et al., 2009
Appetite disorders	Difficulty in swallowing, increase or loss of appetite, preference for sweet foods, different eating mannerisms	Neuropsychiatric Index	34%	Anterior Cingulate Cortex	Hypometabolism	N/A	Ikeda et al., 2002; Zhao et al., 2016
Disinhibition	Inability to withhold unwanted/inappropriate behavior	Neuropsychiatric Index	17%	Cingulate, Frontal Gyri	Atrophy	N/A	Zhao et al., 2016; Serra et al., 2018
Hallucination	Apparent sensual perception of something that is not real	Neuropsychiatric Index, Beliefs About Voices Questionnaire	18–28%	Anterior Right Insula, Occipital Lobe, Temporal Areas	Atrophy, gray matter atrophy	N/A	Holroyd et al., 2000; Ropacki and Jeste, 2005; Blanc et al., 2014; Pezzoli et al., 2022
Delusion	Holding a false belief despite contrary evidence	Neuropsychiatric Index, Peters Delusion Inventory	36%	Right Hippocampus, Temporal Lobes, Left Medial Orbitofrontal Region, Left Occipital Region	Atrophy, reduced cortical thickness, hypometabolism	N/A	Hirono et al., 1998; Geroldi et al., 2002; Ropacki and Jeste, 2005; Serra et al., 2010; Whitehead et al., 2012

Apathy is defined by decreased initiative, diminished affection, social withdrawal, and a lack of emotions (Nobis and Husain, 2018; Lanctôt et al., 2023). It is the most prevalent (19–88%) symptom across all the stages of AD (Starkstein et al., 2006; Marshall et al., 2013; Zhao et al., 2016; Grossman et al., 2021; Mintzer et al., 2021). Apathy is closely associated with AD progression, showing higher rates in AD compared to mild cognitive impairment (MCI) (Lyketsos et al., 2002). In very mild AD, the prevalence of apathy is around 14%, rising to 28% in mild AD, 39% in moderate AD, and 61% in severe AD (Starkstein et al., 2006). Additionally, the severity of apathy worsens as AD progresses (Wetzels et al., 2010). A study reported an average prevalence of 48% in early stages of AD, increasing to 81.9% in the later stages (Grossman et al., 2021). Meanwhile, apathy is closely associated with lower cognitive function (Levy et al., 1998), with potential to increase the likelihood of progression from normal cognition to MCI (Geda et al., 2014). MCI patients with apathy are more likely to develop AD compared to those without apathy (Mendez, 2021; Eikelboom et al., 2022).

Depression is closely related to apathy, as both conditions exhibit a loss of interest, reduced activity, and diminished hedonism, and they can concurrently occur in AD patients. However, they are distinct syndromes (Levy et al., 1998). Depression is primarily attributed to emotional distress and involves symptoms such as sadness, insomnia or hypersomnia, agitation, fatigue, and feelings of worthlessness and hopelessness (Lanctôt et al., 2023). By contrast, apathy is characterized by a lack of motivation and emotional indifference without the emotional distress seen in depression. Despite their differences, clinical differentiation can be challenging due to their potential concurrence. Depression is commonly observed in the preclinical stages of AD, with an overall prevalence of 40% (Zhao et al., 2016; Sáiz-Vazquez et al., 2021). In MCI individuals, the prevalence of depression is about 32% (Ismail et al., 2017; Leung et al., 2021). This prevalence increases in AD patients, reaching 38, 41, and 37% in mild, moderate, and severe dementia, respectively (Ismail et al., 2017; Leung et al., 2021). A decline in depression symptoms in the late stages of AD has also been reported (Botto et al., 2022), possibly attributable to increasingly impaired cognition. MCI individuals with consistent depressive symptoms show a significantly higher rate of progression to AD (31%) compared to those without depressive symptoms (13.5%) (Lee et al., 2012). Additionally, depression in AD is more pronounced in females (Eikelboom et al., 2022).

Anxiety is reported as the third most common NPS in AD (Mendez, 2021). It is characterized by excessive uneasiness or tenseness, often accompanied by avoidance of specific situations or separation anxiety when separated from caregivers (Cummings, 2020). The prevalence of anxiety in AD patients ranges from 26.1 to 70% (Teri et al., 1999; Porter et al., 2003; Zhao et al., 2016; Mendez, 2021). The prevalence rates across mild, moderate, and severe dementia stages are 38, 41, and 37%, respectively, suggesting a relatively stable prevalence across AD stages (Leung et al., 2021). However, some studies reported decreased anxiety in terminal/severe stages AD (Forsell and Winblad, 1997; Chen et al., 2021), possibly due to cognitive impairment (Botto et al., 2022). Anxiety often correlates with other behavioral disturbances, particularly having a high comorbidity with depression at 54% (Teri et al., 1999; Mendez, 2021; Várkonyi et al., 2022). Additionally, recent studies support anxiety as a risk factor for AD and cognitive decline, with a threefold increase in prevalence in AD development (Ma, 2020; Mendez, 2021; Mortby et al., 2017). However, the exact influence of anxiety on AD progression remains uncertain (Breitve et al., 2016).

Agitation and aggression are also common NPS in AD. Agitation is characterized by excessive verbal or motor activity without focus, while aggression involves physical or verbal behaviors intended to cause harm. Both agitation and aggression in AD are linked to increased caregiver distress and earlier institutionalization (Kales et al., 2014). These symptoms become more common and severe as cognitive decline and AD progress (Trzepacz et al., 2013; Choi et al., 2019; Yu et al., 2019). Specifically, 7.4% of MCI patients exhibit aggressive behaviors (Yu et al., 2019) and 70% of AD patients have demonstrated verbal or physical abuse. Studies have commonly reported an increase in aggression and agitation with AD progression (Zahodne et al., 2015). The likelihood of these symptoms significantly increases from Braak stages I/II to Braak stages III/IV (Ehrenberg et al., 2018). However, a reduction in agitation and aggressive behaviors has also been observed, indicating variability in the progression of these symptoms (de Mauleon et al., 2020). Additionally, physical aggression is often associated with delusion, highlighting the comorbidity of these symptoms (Deutsch et al., 1991; Chemerinski et al., 1998). Moreover, although short-term management seems promising, long-term management of agitation and aggression requires further investigation (Ballard and Corbett, 2013) and may require a combination of pharmacological and non-pharmacological approaches (Koenig et al., 2016).

Other NPS in AD encompass a range of behaviors and conditions, including sleep disturbances, circadian rhythm disruptions, prosopagnosia (difficulty recognizing faces), eating disorders (such as hyperphagia or lack of appetite), disinhibition (lack of self-control), hallucination (false sensory perception) and delusion (unshakably untrue belief). The reported prevalence rates for these symptoms are as follows: sleep disturbances (39%), hyperphagia (23%), disinhibition (13%), hallucination (13.4 to 41%) and delusion (51%) (Keene and Hope, 1998; Wilson et al., 2000; Zhao et al., 2016; Linszen et al., 2018). Hallucination and delusion are particularly prevalent in later stages of AD (Craig et al., 2005). The prevalence of delusion increases from 33% in very mild AD to 48% in moderate AD (Chiu and Chung, 2006), while that of hallucination rises from 11.4% in MCI to 28% in severe AD (Ropacki and Jeste, 2005). Sleep disturbances and appetite changes are also more frequently observed in moderate AD compared to mild AD (Keene and Hope, 1998; Kai et al., 2015). By contrast, disinhibition is more attributed to the MCI state (Lyketsos et al., 2002). Overall, these symptoms are linked to increased dementia severity, higher caregiver burden, and more rapid cognitive decline (Wilson et al., 2000; Lyketsos et al., 2002; Linszen et al., 2018).

In short, clinical data highlights the high prevalence of NPS and their worsening over the progression of AD (Liew, 2021). Additionally, NPS are associated with a higher risk of cognitive decline. The presence and progression of NPS underscore the need for targeted interventions to manage these challenging symptoms and improve patient care.

## Pathologies of NPS in AD patients

Human macrostructure (CT, MRI) and microstructure imaging (such as diffusion MRI), as well as functional imaging (fMRI), has been applied to investigate the brain changes related to NPS in AD. Functional imaging studies have revealed several candidate brain structures exhibiting pathological changes in AD patients with NPS. These studies in general intend to associate NPS severity with the neuroimaging deficits, such as hypometabolism, structural changes, and functional connectivity alterations, to identify candidate brain regions contributing to NPS. Based on these studies, commonly identified structures include the anterior cingulate cortex, hippocampus, prefrontal cortex, entorhinal cortex, amygdala, and striatum. Postmortem studies offer even more insight into how amyloid burden, neurofibrillary tangles, and neurotransmitter dysfunction are possibly linked to NPS.

### Apathy related pathology

Apathy in AD is most commonly associated with pathological changes of the anterior cingulate cortex (ACC), which include cortical thinning, atrophy, low fractional anisotropy (FA) scores, amyloid accumulation, hypometabolism, and neurofibrillary tangles (Marshall et al., 2006, 2007; Apostolova et al., 2007; Bruen et al., 2008; Kim et al., 2011; Tighe et al., 2012; Mori et al., 2014; Custo et al., 2024). Other atrophied regions include the prefrontal regions, putamen, caudate nucleus, and the left anterior cingulum white matter tract (Le Heron et al., 2018; Huey et al., 2017). It is hypothesized that apathy is the result of communication failure between the anterior cingulum and other brain structures due to the AD-induced neurological damage (Kim et al., 2011). Overall, apathy is correlated with more cortical amyloid burden (Marshall et al., 2013; Mori et al., 2014) in the early stages of AD. However, this may be independent of cognitive changes (Johansson et al., 2022). Similarly, early tau aggregation in the trans-entorhinal region is associated with more severe apathy (Yasuno et al., 2020). Besides the dorsal anterior cingulate cortex and ventral striatum (Le Heron et al., 2017), the basal forebrain (Sperling et al., 2023) is also associated with apathy in neurodegenerative diseases. Neuroimaging data have revealed abnormality of basal forebrain activity in apathy (Sperling et al., 2023). The observation that cholinesterase inhibitors could improve apathy symptoms in AD patients further suggests that the basal forebrain, the major structure hosting central cholinergic neurons, may be involved in apathy in AD. Moreover, our recent studies have reported that glutamatergic neurons in the medial septum (MS) of the basal forebrain encode negative-valence sensory events and drive avoidance behavior (Zhang et al., 2018a,2018b), while its GABAergic neurons, specifically those expressing somatostatin, encode positive-valence events and drive appetitive behavior (Shen et al., 2022). Both of these neurons may exhibit deficits in AD-apathy (Zhang et al., 2023). Furthermore, in both human AD patients and animal models, MS has been considered as one of the earliest brain structures affected by neurodegeneration (Auld et al., 2002; Fujishiro et al., 2006; Wu et al., 2021), suggesting that functional impairments of MS may contribute to apathy. Meanwhile, postmortem studies have shown dopaminergic dysfunction in circuits involving the basal ganglia, anterior cingulate, and frontal cortices (Lai et al., 2011; Martorana and Koch, 2014).

### Depression related pathology

There are converging pathological changes in the brains of depression patients without and with AD. These include alterations in the monoaminergic system, changes in glutamatergic synaptic transmission, neuro-inflammation, alterations in the hypothalamic-pituitary-adrenocortical (HPA) axis, changes in brain-derived neurotrophic factor (BDNF), and hippocampal atrophy (Dolotov et al., 2022). In AD patients with depression, reductions in monoamines, including dopamine, serotonin, and noradrenaline, have been reported (Nazarali and Reynolds, 1992). These changes have long been associated with depression (Delgado, 2000).

However, depression in AD is more specifically linked to deficits in certain brain regions and AD-related pathological changes. Imaging studies have generally observed that reductions in gray matter volume and cortical thinning are associated with the frequency and severity of depressive symptoms in AD (Brommelhoff and Sultzer, 2015). The affected areas include the anterior cingulate, prefrontal cortex, entorhinal cortex, parietal cortex, and basal ganglia (Hirono et al., 1998; Holthoff et al., 2005; Lee et al., 2010, 2012; Son et al., 2013; Zahodne et al., 2013; Lebedeva et al., 2014; Chen et al., 2021). Additionally, gray matter atrophy in the hippocampal and striatal regions is associated with depression in AD, indicating their involvement in AD-related depressive symptoms as well (Morra et al., 2009; Brommelhoff et al., 2011). Functional connectivity studies have shown that in AD patients with depression, there is increased connectivity between the amygdala and orbitofrontal cortex and decreased connectivity between the amygdala and medial prefrontal cortex (mPFC) (Guo et al., 2018). Beyond macrostructural changes, high amyloidopathy is associated with an increased risk of depression among MCI patients (Lopez-Jimenez et al., 2019; Youn et al., 2019). Postmortem studies indicate that white matter hyperintensity in the bilateral occipital area (Desmarais et al., 2021), chronic cerebral hypoperfusion in the orbitofrontal cortex and dorsolateral prefrontal cortex (Sinclair et al., 2023) and neurofibrillary tangle (NFT) burden in Braak stage I/II (Ehrenberg et al., 2018) are correlated with AD-depression. Other pathological correlations include a reduction in hippocampal serotonergic 5-HT1A receptors (Lai et al., 2011), abnormal fatty acids in the prefrontal cortex and hippocampus suggesting demyelination (Nihonmatsu-Kikuchi et al., 2013), and a reduction in antioxidant enzymes in the hippocampus and anterior cingulate cortex (Luo et al., 2022). Additionally, a lower number of noradrenergic and cholinergic neurons has been reported to be associated with depression in AD (Förstl et al., 1992).

While it remains a challenge to understand the intricate relationship between depression and AD, the high comorbidity of AD and depression suggests some shared underlying pathological changes. The pathological changes observed in both AD-depression and non-AD depression may shed light on potential common underlying mechanisms that can inform treatment strategies.

### Anxiety related pathology

Anxiety in AD is primarily linked to the hippocampus, entorhinal cortex, amygdala, and prefrontal cortex. Related pathological changes include decreased volume, atrophy, and hypometabolism in the said regions (Hashimoto et al., 2006; Poulin et al., 2011; Tagai et al., 2014; Mah et al., 2015; Mohamed Nour et al., 2021; Chen et al., 2021). Higher amyloid-beta (Aβ) burden is associated with more severe anxiety in AD patients (Goukasian et al., 2019; Lopez-Jimenez et al., 2019), which is also the case in cognitively normal elders (Donovan et al., 2018). In early AD stages, development of anxiety is more associated with Aβ accumulation but is independent of cognitive decline (Johansson et al., 2022). In cognitively normal elder individuals, anxiety is found to be the highest in APOEε4 carriers with subcortical rather than cortical amyloidosis (Hanseeuw et al., 2020). Postmortem investigations have further associated AD-anxiety to high NFT and Aβ burden in limbic regions, anterior cingulate and entorhinal cortex, as well as more serotonergic transporters in the temporal cortex (Tsang et al., 2003; Ehrenberg et al., 2018; Das, 2023).

### Agitation and aggression related pathology

Studies on agitation and aggression in AD have identified various neuronal and functional changes, including atrophy, low fractional anisotropy scores, increased functional connectivity, amyloid accumulation, and hypometabolism in regions such as the amygdala, anterior cingulate, hippocampus, frontal insula, and frontal cortex (Tekin et al., 2001; Trzepacz et al., 2013; Mendez, 2021). Frontal lobe dysfunction has been proposed to be associated with agitation in AD. The severity of frontolimbic atrophy is correlated with the severity of aggression, with neurodegeneration of the anterior salience network possibly leading to a reduced ability to regulate behavior (Trzepacz et al., 2013). NFT burden in the orbitofrontal and anterior cingulate cortex correlates with this symptom in AD (Tekin et al., 2001). Tau pathology shows a stronger correlation with aggression compared to amyloid pathology in AD (Carrarini et al., 2021). Additionally, the decreased glucose metabolism in the frontal lobe and increased sensitivity to noradrenergic signaling have been associated with agitation and aggression in AD (Carrarini et al., 2021). Postmortem examinations have further revealed that lowered choline acetyltransferase (ChAT) activity in the frontal and temporal cortex is correlated with AD-agitation and AD-aggression (Minger et al., 2000; Garcia-Alloza et al., 2005). Serotonergic deficits in the hippocampus may also play a role, as serotonin re-uptake (5-HTT) sites are preserved or upregulated in aggressive compared to non-aggressive patients and 5-hydroxyindoleacetic acid (5-HIAA) levels inversely correlate with agitation scores (Lai et al., 2011; Vermeiren et al., 2014). Meanwhile, traumatic brain injury, smoking, and phosphorylated TAR DNA-binding protein 43 (TDP-43) may contribute to aggression and agitation in AD (Sennik et al., 2017).

### Other NPS-related pathology

Neuroimaging and postmortem studies have also been conducted to examine other neuropsychiatric symptoms. Atrophy of the right hippocampus and temporal lobes (Geroldi et al., 2002; Serra et al., 2010), reduced cortical thickness of the left medial orbitofrontal region (Whitehead et al., 2012), and hypometabolism of the left occipital region (Hirono et al., 1998) have been found to be correlated with delusion. Hallucinations are associated with changes such as atrophy in the anterior right insula (Blanc et al., 2014) and occipital lobe (Holroyd et al., 2000), and gray matter atrophy in occipital and temporal areas in the case of visual hallucinations. Right middle frontal gyrus hyperperfusion and decreased cerebrospinal fluid (CSF) melatonin are associated with sleep disturbances (Liu et al., 1999; Ismail et al., 2009), hypometabolism in the anterior cingulate cortex with appetite disturbances (Hu et al., 2002), and atrophy of the cingulate and frontal gyri with disinhibition (Serra et al., 2010).

In the above research, imaging studies have been particularly valuable for understanding neural substrates of NPS, revealing a wide range of candidate brain structures with pathological changes associated with each symptom. Most of the affected structures–hippocampus, amygdala, frontal cortex, and anterior cingulate cortex—are known to play a role in emotional regulation and cognition. In particular, the anterior cingulate cortex and frontal cortex manifest pathological changes across all symptoms covered in this review, marking them potentially critical regions for most AD-associated NPS. Amyloid and tau pathology is not only correlated with more frequent and severe cases of NPS (Goukasian et al., 2019; Lopez-Jimenez et al., 2019; Youn et al., 2019; Babulal et al., 2022) but also may provide early markers for AD-associated NPS in the form of CSF levels of total tau (t-tau) and Aβ-42 (Babulal et al., 2022). However, human imaging studies on AD-associated NPS are yet constrained by several limitations. While imaging can reveal pathological changes that occur in more advanced stages of AD, NPS often emerge in the preclinical stages before such features are identifiable. The lack of well-defined biomarkers further hampers the ability to track the trajectory of AD-associated NPS. For example, there is disagreement on when Aβ accumulation initially starts (Chen et al., 2021). Studies on the increases of CSF t-tau and Aβ42 in MCI patients have examined NPS in general rather than individual specific symptoms. Other proposed early markers of AD, such as a hyperactive anterior cingulate cortex (Yuan et al., 2022), have not been investigated thoroughly in the context of AD-associated NPS. Moreover, most longitudinal studies on this topic have been empirical, with a scant number of them providing quantitative analysis of the symptom progression. Existing studies also overlook the frequent comorbidity of NPS features, rather focusing on isolated symptoms. The lack of universal NPS assessments is also an obstacle, translating into ambiguous standards of data validity. Although the Diagnostic and Statistical Manual of Mental Illnesses (DSM V) lays out criteria for diagnosis, it still relies on individual interpretation and is subject to revision. Finally, technical limitations prevent an in-depth understanding of molecular or genetic changes underlying NPS in humans. Considering the limitations, animal models may be indispensable as to provide more insights into the etiology of AD-associated NPS.

#### NPS in LBD and FTD

NPS are also prevalent across other forms of dementia, including Lewy body dementia (LBD) and frontotemporal dementia (FTD). Common symptoms in LBD and FTD include delusion, hallucination, aggression, depression, anxiety, euphoria, apathy, disinhibition, appetite changes, and sleep disturbances (van de Beek et al., 2022; Wright et al., 2023). LBD, in particular, often presents with higher severity and prevalence of NPS than AD, with pronounced symptoms of apathy, hallucination and delusion (Ballard et al., 2001; Leung et al., 2021; Liu et al., 2021). FTD shares many of these NPS features but frequently exhibits anxiety, apathy and disinhibition as core symptoms (Tyrrell et al., 2020; Collins et al., 2023). Notably, studies show that most LBD patients experience at least one NPS episode, with symptoms often fluctuating or recurring (Vik-Mo et al., 2020), while FTD also exhibits fluctuating NPS patterns, often prominent in both early and late stages (Morrow et al., 2024).

Pathologically, while AD and LBD share some similarities—such as amyloid-beta plaque accumulation—LBD typically has less tau pathology, a hallmark of AD (Geng et al., 2024). NPS manifestation in LBD is closely linked to Lewy Body Braak staging, particularly associated with hallucinations, while in AD, NFTs are more often linked to depression and agitation (Gibson et al., 2022). In FTD, pathology is frequently marked by TDP-43 and tau protein deposits (Shaw et al., 2024). When LBD and AD co-occur, an additive effect on symptom severity is often noted (Gibson et al., 2022). Given the potential overlap in pathologies among these dementias, comparing NPS across AD, FTD, and LBD can provide insights into shared mechanisms, offering pathways for more nuanced, multi-faceted treatments.

## NPS-like behavioral deficits in AD mouse models

**FIGURE 2:**
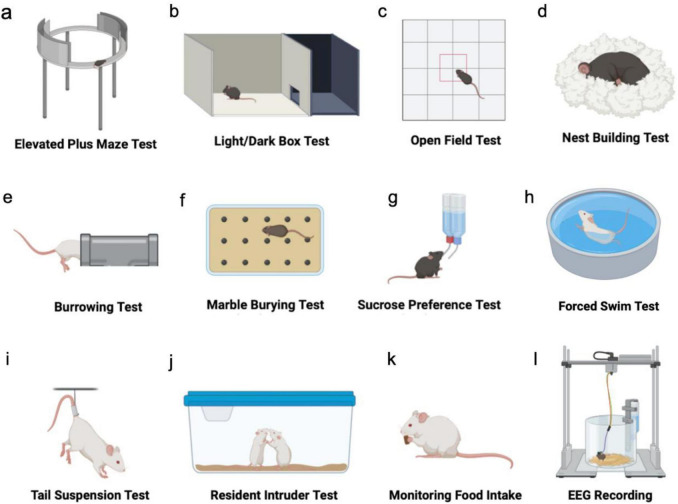
Testing typical NPS-like behavior in mouse models. **(a–c)** Tests to assess anxiety-like behaviors. **(d–f)** Tests to assess apathy-like behaviors. **(g–i)** Tests to assess depression-like behaviors. **(j)** Test of aggressive behavior. **(k)** Test of hyperphagia. **(l)** Test of sleep disturbances.

Mouse models provide us a great opportunity to examine the impacts of AD-related pathology on NPS-like behavioral deficits. Currently, various AD mouse models have been developed with pathological progression, molecular changes, and cognitive functions thoroughly investigated. However, the use of AD mouse models to specifically study AD-associated NPS is mostly lacking. The high prevalence and significant impacts of NPS in patients highlight the urgent need to investigate AD-associated NPS using rodent models. Specific brain regions and circuits, such as the orbital frontal cortex and anterior cingulate cortex, can be correlated with NPS-like manifestations. Additionally, exploring the intersection of genetic risk factors for both AD and NPS could yield significant insights (Table 2 and Figure 2).

**TABLE 2:** NPS-like behaviors and pathologies in AD mouse models.

NPS category	Testing methodology	Mouse model(s)	Common pathological changes and related brain structures	Corresponding citations
Apathy-like behaviors	Nest Building Test, Burrowing Test, Marble Burying Test, Sucrose Preference Test	5xFAD, APP/PS1, P301S, 3xTg	High levels of amyloid-beta in prefrontal cortex and hippocampus, dopaminergic neuron degeneration in substantia nigra and VTA	Huang et al., 2016; Pardossi-Piquard et al., 2016; Vorobyov et al., 2019; Sun et al., 2020; Keszycki et al., 2021; O’Leary and Brown, 2024
Anxiety-like behaviors	Elevated Plus Maze Test, Light/Dark Box Test, Open Field Test, Marble Burying Test	5xFAD, APP/PS1, 3xTg, P301S, THY-Tau22, TgF433, ICV-STZ Injection, APOE4	Neuroinflammation in hippocampus and amygdala, decreased GABA synthesis, amyloid-beta accumulation in hippocampus, altered glutamatergic activity	Schindowski et al., 2006; Takeuchi et al., 2011; Pentkowski et al., 2018; Dong et al., 2020; Hunsberger et al., 2021; Roy et al., 2022; Taxier et al., 2022; Garcia et al., 2023; Szabó et al., 2023
Depressive-like behaviors	Nest Building Test, Burrowing Test, Marble Burying Test, Sucrose Preference Test, Forced Swim Test, Tail Suspension Test	5xFAD, APP/PS1, 3xTg, APOE4, 6xTg, P301S	High levels of amyloid-beta in hippocampus and cortex, neuroinflammation, tau pathology, neurotransmitter changes in serotonin and dopamine	Romano et al., 2015; Nie et al., 2017; Gao et al., 2018; Nyarko et al., 2019; Dong et al., 2020; Locci et al., 2021; Zhang J. et al., 2021; Pierson et al., 2022; Tian et al., 2023; Kim et al., 2023; Robinson et al., 2024
Aggressive behaviors	Resident Intruder Test	5xFAD, APP/PS1, Tg2576, TAPP, 3xTg, P301L	Amyloid plaques in nucleus accumbens, cortical, hippocampal and amygdala regions, increased tau pathology, altered neuronal activity in mPFC	Alexander et al., 2011; Jul et al., 2015; Fernández-Pérez et al., 2020; Zhang et al., 2022; Warfield et al., 2023; Kosel et al., 2024
Sleep disturbances	EEG recording	5xFAD, APP/PS1, TgCRND8, 3xTg, P301S	Amyloid plaques in hippocampus, GABA-positive astrogliosis in sleep-promoting regions, inflammation in brain and plasma	Colby-Milley et al., 2015; Cushing et al., 2020; Holth et al., 2017; Minakawa et al., 2017
Disinhibition	Open Filed Test	APP^SAA^	Alteration of lipids, microglia transcriptomic changes, intracellular amyloid content	Xia et al., 2022

### Apathy-like behavior and related pathology in AD mouse models

Various AD mouse models, including those with amyloid precursor protein (APP) related and Tau pathologies, exhibit significant apathy-like behaviors. Two commonly used behavioral assays to imply apathy-like deficits are the nest building and sucrose preference test, with nest building reflecting self-care and sucrose preference serving as an indicator of reward perception. The L66 mouse line, which overexpresses the full-length human tau with double mutations (P301S/G335D), demonstrates reduced nest building and sucrose preference at 5-6 months old, suggesting apathy-like deficits (Melis et al., 2015; Robinson et al., 2024). Such deficits are similarly observed in P301S mice, particularly through notable changes in nest building (Sun et al., 2020). Furthermore, the 5xFAD mouse, which overexpresses human APP with three mutations along with human presenilin 1 (PS1) harboring two mutations, begins to show significant apathy-like behaviors from six months of age, as measured by nest-building, burrowing, and marble burying (Keszycki et al., 2021; O’Leary and Brown, 2024). In addition, APP/PS1 mice, which are double transgenic mice expressing a chimeric mouse/human APP and a mutant human presenilin 1, display increased apathy-like behaviors compared to wild-type mice (Pugh et al., 2007; Huang et al., 2016). The 3xTg-AD mouse bearing human mutations of APP, PS1 and tau also develop early apathy-like deficits, marked by a decrease in spontaneous motor activity (Pardossi-Piquard et al., 2016). Moreover, an interval timing behavioral test in this model reveals a sex difference in that female mice show delayed anticipatory respondings and lower response amplitudes compared to males, suggesting differential incentive motivation between sexes in these AD mice (Gür et al., 2019).

The above observations suggest that apathy-like behaviors might be associated with both Aβ and Tau pathologies. In 5xFAD mice, increased apathy-like behaviors such as those assessed by nest building and marble burying are associated with soluble Aβ-42 and plaques in the prefrontal cortex (PFC) and hippocampus (Keszycki et al., 2021). Additionally, the presence of amyloid beta in the striatum of APP-NLGF mice (containing the Swedish KM670/671NL mutation, the Iberian I716F mutation and the Artic E693G mutation) is inversely correlated with motivational levels (Hamaguchi et al., 2019). Similarly, in 3xTg mice, Aβ pathology observed in the hippocampus, amygdala, and entorhinal cortex has been suggested to contribute to apathy-like behavior (Pardossi-Piquard et al., 2016; Bourgeois et al., 2018). Apathy-like behavior can be observed in Tau models as well (Robinson et al., 2024); however, more specific correlation with pathology requires further investigations. Neurodegeneration in brain regions involved in depression and apathy (Krashia et al., 2022), particularly that of dopaminergic neurons in the substantia nigra and ventral tegmental area (VTA), along with decreases in dopamine transporters in the cortex and hippocampus of 5xFAD mice, plays a significant role in apathy and depression-like symptoms (Vorobyov et al., 2019). Early degeneration in the nucleus accumbens in the APP/PS1 mouse model is associated with an Aβ-linked increase in membrane excitability in medium spiny neurons and a reduction in glycinergic synaptic transmission, which may link to apathy (Fernández-Pérez et al., 2020). Furthermore, APP/PS1 mice exhibit significant hyperactivity in the dorsal raphe nucleus during reward-seeking and motivational actions (Sakurai et al., 2020), indicating complex interactions between Aβ pathology and neural circuitry affecting behavior. In general, Aβ plaques in the striatum are linked to lower levels of motivation and dopamine transporter (DAT) expression (Hamaguchi et al., 2019).

### Anxiety-like behavior and related pathology in AD mouse models

Anxiety-like behaviors can be assessed using various behavioral tests, including the open field test (OFT), elevated plus maze (EPM), light-dark box, social interaction, and novelty-suppressed feeding. Each test provides insight into a distinct aspect of anxiety-related behavior. In 5xFAD mice, anxiety-like behaviors typically begin at 5 months old, with males showing more profound symptoms (Angel et al., 2020; Dong et al., 2020; Li et al., 2022). Increased social anxiety in novel environments is also noted using an odor investigation paradigm (Kosel et al., 2019). However, some other studies report no increase or even a reduction in anxiety-like behaviors (Jawhar et al., 2012; Schneider et al., 2014; Garcia et al., 2023), and different tests within the same study can yield varied results (O’Leary and Brown, 2024). In APP/PS1 mice, females exhibit anxiety-like behavior correlated with memory impairment at an earlier age compared to control and male groups (Hunsberger et al., 2021), while males also show increasing anxiety severity with age (Huang et al., 2016). Social defeat stress and poor sleep quality are linked to anxiety-like behavior (Yao et al., 2023). Nonetheless, some studies report no significant differences in anxiety-like behaviors in this model (Pugh et al., 2007; Locci et al., 2021). The widely used 3xTg mouse model, which contains three mutations associated with familial Alzheimer’s disease, can display anxiety-like behaviors through behavioral paradigms such as the OFT and EPM test as early as 2 months of age (Paciello et al., 2021). In the fox-odor container test, 3xTg mice exhibit increased predator odor-induced anxiety, which is detectable at 2 months of age (i.e., before the onset of AD pathology) and is more pronounced in aged females than males (Szabó et al., 2023). In P301S mice, however, reduced anxiety-like behaviors are observed, even before tau pathology appears (Takeuchi et al., 2011). For example, P301S mice have been observed to spend relatively more time in open arms of EPM or in the center area of OFT compared to wildtype control, while they may exhibit sexual dimorphism (Dumont et al., 2011; Sun et al., 2020; Watt et al., 2020). Other animal models, including APP-NLGF knock-in mice (Pugh et al., 2007), APOE4 (relative to APOE3) mice (Siegel et al., 2012; Taxier et al., 2022), TgF433-AD rats (Pentkowski et al., 2018), Wistar rats injected with intracerebroventricular streptozotocin to induce sporadic AD (Roy et al., 2022), and THY-Tau22 (which expresses human 4-repeat tau mutated at sites G272V and P301S under a Thy1.2-promotor) (Schindowski et al., 2006), also display increased anxiety-like behaviors. It is worth noting that a study using APP-NLGF knock-in mice has found conflicting anxiety profiles as shown by the increased thigmotaxis in OFT (i.e., anxiogenic) but reduced closed-arm time in EPM (anxiolytic) (Pervolaraki et al., 2019). Altogether, while anxiety-like behaviors are prevalent across various AD models, the onset age, severity, and sex dependence vary greatly. Conflicting results within and across studies highlight the complexity of anxiety-like phenotypes in these models, emphasizing the need for comprehensive behavioral tests to understand underlying mechanisms.

Investigations into the neurobiological underpinnings of anxiety-like behaviors in AD models have suggested multiple factors, such as Aβ deposition, Tau pathology, neuroinflammation, and neuronal or neurotransmitter changes. First of all, the presence of amyloid beta accumulation is closely linked to anxiety-like phenotypes. In 5xFAD mice, early-life stress such as maternal separation results in enhanced Aβ deposition in the anterior cingulate cortex and basolateral amygdala (Garcia et al., 2023). Caspase-6 knockout in 5xFAD mice on the other hand has reduced Aβ pathology and improved performance in anxiety assessment compared to 5xFAD alone (Angel et al., 2020). In APP/PS1 mice, anxiety-like behavior is associated with high levels of soluble Aβ in the hippocampus (Gao et al., 2018). An increased amount of Aβ deposition and decreased expression of a proteolytic enzyme responsible for the degradation of intracellular Aβ have been found in aged APP/PS1 mice, particularly in the dorsal hippocampus (Huang et al., 2016). Anxiety-like behavior can be induced in rats through intracerebroventricular streptozotocin (ICV-STZ) injections, which is accompanied by diffuse amyloid plaques in the hippocampus, enlargement of ventricles, and spine loss in the dentate gyrus (Roy et al., 2022). In 3xTg mice, widespread Aβ accumulation in the hippocampus, amygdala, olfactory bulb, and piriform cortex correlates with manifestation of anxiety-like behavior (Paciello et al., 2021; Tournissac et al., 2021; Szabó et al., 2023). Homozygous APP-NLGF mice that exhibit anxiety-like behaviors have widespread Aβ deposition at 6 months of age (Whyte et al., 2018). More specifically, overexpression of insoluble Aβ may drive alterations in gamma oscillations and glutamatergic gene expression in the prefrontal cortex, potentially underlying changes in anxiety-related behaviors (Pervolaraki et al., 2019). Furthermore, the tauopathy model THY-Tau22 has phosphorylated tau expression correlated with hippocampal dysfunction and anxiety-like phenotypes (Schindowski et al., 2006).

Neuroinflammation may also be a contributing factor. Caspase-6 knockout in 5xFAD mice results in concurrent reductions in anxiety-like behavior and neuroinflammatory indicators compared to 5xFAD alone (Angel et al., 2020). Increased neuroinflammatory markers such as interleukin-10 (IL-10) in the hippocampus and ionized calcium binding adaptor molecule 1 (IBA1) in the anterior cingulate are associated with anxiety-like behaviors in 5xFAD mice, which are further exacerbated by early-life stress (Garcia et al., 2023). The hippocampus displays microglial activation, elevated IL-6 and tumor necrosis factor alpha (TNF-α), as well as neurotrophic factor signaling disturbances in APP1/PS1 mice with anxiety-like phenotypes (Gao et al., 2018). Hippocampal neuroinflammation and anxiety-like behavior are also co-manifested in 3xTg mice (Paciello et al., 2021). A study examining glycogen synthase kinase-3β (GSK-3β) overexpressing mice, a genetic model for neurodegeneration, further suggests that neuroinflammation in the ventral rather than dorsal hippocampus contributes to anxiety-like behavior (Fuster-Matanzo et al., 2011). In P301S mice with anxiety-like phenotypes, inflammation markers appear to differ between sexes: males express more monokines induced by interferon gamma (MIG), TNF-α, IL-13, and IL-10 than females (Takeuchi et al., 2011; Sun et al., 2020). Additionally, microglial activation is evident before the appearance of tau pathology (Dumont et al., 2011).

Neurotransmitter changes can be associated with anxiety-related behavioral changes. In 5xFAD mice, impeded neuronal GABA synthesis has been found in the hippocampus (Andersen et al., 2021). A study on effects of positive and negative allosteric modulators (PAM and NAM, respectively) of α5 GABAA receptors has shown that 10-day treatment of NAM reduces the level of anxiety in six-month-old 5xFAD male mice, but without an effect in female transgenic mice, suggesting sex-specific changes in GABA signaling (Aranðeloviæ et al., 2021). In addition, NAM treatment increases expression of GABA receptor alpha subunit GABRA2 in the prefrontal cortex of male transgenic mice, while PAM treatment decreases GABRA5 in both sexes. Regarding glutamatergic signaling, a decrease in anxiety-like behaviors in 5xFAD mice older than 5 months is accompanied by an age-dependent reduction in mGluR5 binding availability in the hippocampus, striatum, cerebellum and cortex (Lee et al., 2019). There is evidence that anxiety-like phenotypes may be related to melatonin deficiency, as treating 3xTg mice with melatonin not only ameliorates behavioral deficits but also downregulates the glutathione S-transferase P 1 (GSTP1) protein in the hippocampus (Nie et al., 2017). Furthermore, with respect to dopaminergic signaling, a reduction in anxiety-like behaviors and an increase in locomotor activity in 3xTg mice are accompanied by an increase in postsynaptic D2 dopamine receptors in the striatum and in D2-autoreceptors in substantia nigra/VTA cell bodies (Gloria et al., 2021).

Neuronal loss associated with anxiety-related behavioral changes occurs in several AD mouse models. In 5xFAD mice, selective neuronal loss is found in cortical layer 5 (L5), without affecting the overall neuron number in the total frontal cortex and hippocampus, and this correlates with the reduced anxiety-like behavior (Jawhar et al., 2012). Another study reports that a significant loss of parvalbumin and somatostatin inhibitory neurons in the ventral hippocampus, which results in excitation/inhibition (E/I) imbalance, leads to anxiety-like behavior (Li et al., 2022). In aged APP/PS1 mice with anxiety-like phenotypes, there are decreases in hippocampal volume and neuronal number (Huang et al., 2016). In 3xTg mice, a reduction in cortical cholinergic fibers signifying cholinergic neuron loss has been observed (Várkonyi et al., 2022). As for other neuronal changes, a decline in hippocampal CA2 activity correlates with anxiety-related behavior in female APP/PS1 mice (Hunsberger et al., 2021). In these transgenic mice, optogenetic stimulation of the circuit connecting the posterior basolateral amygdala (BLA) to calbindin-positive neurons in the ventral hippocampal CA1 alleviates anxiety-like behavior, suggesting hypoactivity of the calbindin-positive neurons (Pi et al., 2020). Additionally, optogenetically activating GABAergic neurons in the ventral tegmental area (VTA) reduces anxiety induced by social defeat stress through improving sleep quality (Yao et al., 2023). In APOE knockout mice expressing human APOE4, enhanced measures of anxiety are associated with reduced microtubule-associated protein 2 (MAP2)-positive neuronal dendrites in the central nucleus of the amygdala (Robertson et al., 2005).

Other factors that may influence anxiety-like behaviors in AD mouse models include oxidative stress and ovarian hormones. The P301S mice with anxiety-like phenotypes display decreased activity of mitochondrial enzymes involved in reactive oxygen species formation and oxidative stress prior to tauopathy (Dumont et al., 2011). Female 5xFAD mice that express human APOE4 (E4FAD) exhibit increased anxiety-like behavior relative to E3FAD, and ovariectomy (OVX) in E3FAD mice increases anxiety to a similar level to intact and OVXed E4FAD mice, suggesting a protective role of ovarian hormones against anxiety-like phenotypes in E3FAD but not E4FAD mice (Taxier et al., 2022).

### Depressive-like behavior and related pathology in AD mouse models

Behavioral changes in various mouse models reveal a complex interplay of genetic and environmental factors linking AD to depressive-like behavior. In 5xFAD mice, compelling depressive-like phenotype, such as increased immobility in the tail suspension test (TST), has been observed starting from 5 months of age (Patel et al., 2014; Dong et al., 2020; Locci et al., 2021; Chen et al., 2024). Environmental factors, including postnatal maternal separation and early-life stress, can also induce expression of depressive-like behaviors at 4 months, as demonstrated by open field and novel object recognition tests (Bachiller et al., 2022). In the L66 tauopathy model, anhedonia is evident as shown by the decreased sucrose preference (Robinson et al., 2024). Depressive-like behaviors are also evident in pharmacologically induced AD models, such as those involving intracerebroventricular injection of Aβ oligomers (Ledo et al., 2013; Torrisi et al., 2019). APP/PS1 transgenic mice show depressive-like behaviors, demonstrated by reduced sucrose preference, increased immobility in the TST and forced swimming test (FST), as well as social withdrawal before Aβ plagues are detected (Gao et al., 2018; Martín-Sánchez et al., 2021). Similarly, the APP-NLGF model recapitulates depression-like phenotypes, including reduced social interaction and increased immobility (Dong et al., 2020; Locci et al., 2021). In the 3xTg model, depressive-like phenotypes are evident as early as 4 months of age in FST, TST, and splash test (Romano et al., 2015; Nie et al., 2017; Várkonyi et al., 2022). Furthermore, APOE4-targeted replacement mice, carrying the human APOE4 allele (a known strong risk factor for developing late-onset AD), display significant depressive-like behaviors compared to APOE3 counterparts, especially under stress conditions (Fang et al., 2021; Zhang J. et al., 2021; Li et al., 2024). Other mouse models, including 6xTg (with tau and Aβ pathologies) and P301L-tau, also demonstrate depression-like behaviors (Locci et al., 2021; Pierson et al., 2022; Kim et al., 2023).

The presence of Aβ is closely associated with depression-like behaviors in AD animal models. Research shows that intracerebroventricular injection of soluble Aβ-42 in wildtype rats and mice induces depressive-like behavior, as shown by increased immobility in FST (Colaianna et al., 2010; Ledo et al., 2013). In APP/PS1 mice having been exposed to chronic mild stress, high levels of soluble Aβ in the hippocampus correlate with the manifestation of depressive-like behaviors (Gao et al., 2018). Similarly, in 6xTg mice, a significant positive correlation has been found between depression-like behavior and the levels of Aβ and phosphorylated tau in the cortex and hippocampus (Kim et al., 2023). Neuroinflammation appears to play a crucial role in these depressive-like phenotypes, particularly in regions such as the hippocampus, prefrontal cortex, anterior cingulate cortex and basolateral amygdala (Garcia et al., 2023). Such inflammation, marked by increased cytokine levels, microglial activation, and oxidative stress, may result from Aβ accumulation (Boza-Serrano et al., 2018; Rejc et al., 2022). Supporting this notion, wildtype mice injected with Aβ oligomers exhibit both depressive-like behaviors and microglial activation in the cortex and hippocampus, as well as aberrant TNF-α signaling (Ledo et al., 2013; Ledo et al., 2016), in agreement with the existence of common pathological features between AD and major depressive disorder (Torrisi et al., 2019). These mice also exhibit pruning of spines and silencing of excitatory synaptic transmission in the nucleus accumbens (Guo et al., 2022). Stress conditions such as early life stress (ELS) can aggravate neuroinflammation, further contributing to AD-related depressive-like behaviors (Gao et al., 2018; Bachiller et al., 2022; Li et al., 2024). Similar to amyloid pathology models, positive correlations between depressive-like behavior and tau pathology in the hippocampus and cortex and associated neuroinflammation have been observed in Tau pathology models such as 6xTg and THY-Tau22 (Schindowski et al., 2006; Kim et al., 2023).

Neurotransmitter and neuromodulator changes have been reported in conjunction with depressive-like behaviors in AD mouse models. The deficiency of monoamines such as dopamine and serotonin, a well-known contributor to mood dysregulation, is a strong indicator of altered cellular function of the earliest stages of AD pathology (Nyarko et al., 2019; Romano et al., 2015). Dopaminergic neuron degeneration is present in the substantia nigra and VTA of 5xFAD mice, with decreased dopamine transporter levels in the hippocampal dentate gyrus and secondary motor cortex (Vorobyov et al., 2019). Decreased serotonin release and serotonergic fiber density are observed especially in the hippocampal region of 5xFAD mice (Tian et al., 2023), a feature also seen in depressive rats following intracerebroventricular injection of soluble Aβ-42 (Colaianna et al., 2010). Overexpression of P301L tau in the dorsal raphe nucleus of wildtype mice leads to depressive-like behaviors and hyperexcitability of serotonin neurons (Pierson et al., 2022). A decline in stress-induced uptake of serotonin in the hippocampus and prefrontal cortex of mice carrying APOE4 relative to APOE3 is associated with an increase in stress-induced depressive-like behavior (Fang et al., 2021). Regarding altered neuronal/synaptic activity, a reduced glutamatergic projection from ACC to the ventral hippocampal CA1 area is suggested to contribute to depressive-like symptoms in 3-month-old 5xFAD mice (Chen et al., 2024). A decrease in levels of hippocampal and cortical mGluR5, vesicular glutamate transporter 1 (VGLUT1) and other glutamatergic signaling related proteins is correlated with depression-like behaviors in 6xTg and 3xTg-AD mice (Cassano et al., 2012; Kim et al., 2023). Additionally, in mice carrying APOE4, stress induces a loss of GABAergic neurons in the prefrontal cortex and hippocampal dentate gyrus, which may contribute to an increased risk of depression (Zhang J. et al., 2021). Other factors that contribute to AD-related depressive behaviors include a deficiency in melatonin (Nie et al., 2017), impaired glucose metabolism, mitochondrial dysfunction and reduced levels of adenosine triphosphate (ATP) (Li et al., 2024).

### Aggressive behavior and related pathology in AD mouse models

The rate of injurious behavior is increased in male 5xFAD mice when housed together, compared to wildtype or mixed genotype groups (Kosel et al., 2021). Similarly, in a number of Aβ and tau models, such as APP/PS1 (Pugh et al., 2007; Huang et al., 2016; Morgastrern et al., 2018), Tg2576 (Alexander et al., 2011), 3xTg (Zhang et al., 2022) and rTg4510 (Jul et al., 2015), males exhibit significant aggressive behavior compared to wild-type mice, as assessed by the resident-intruder test. In some studies, aggressivity is only observed during specific phases of circadian rhythm (Bergamini et al., 2022; Warfield et al., 2023), suggesting a complex interaction between circadian rhythm and aggressive behavior.

In male mouse models of amyloidosis, such as APP/PS1 and TASD41 (hAPP751 with the London V717I9/Swedish double mutation K670M/N671L), aggressive behavior correlates with a progressive increase in amyloid plaque size and density in cortical, hippocampal, and amygdala regions (Morgastrern et al., 2018). The nucleus accumbens (NAc), known for its role in aggression and motivation (Salgado and Kaplitt, 2015), shows increased intraneuronal Aβ accumulation and extracellular amyloid deposits in the APP/PS1 model (Fernández-Pérez et al., 2020). This is associated with an increase in membrane excitability in medium spiny neurons and a decrease in glycinergic synaptic transmission, suggesting a link between NAc dysfunction and aggression (Fernández-Pérez et al., 2020). Additionally, in 3xTg mice, increased excitability of thalamus-projecting pyramidal tract (PT) neurons in the mPFC, which could be attributed to decreased Kv6.3 channels in these neurons, may underlie the increased irritability and aggressivity (Zhang et al., 2022). In 5xFAD mice, aberrant local field potential (LFP) activity has been observed in the mPFC during exposure to social olfactory stimuli, consistent with the notion that increased social anxiety leads to aggressivity (Kosel et al., 2019, 2024). As for tauopathy models, increased aggressive or agitative-like behavior has been associated with the progression of tau pathology (Jul et al., 2015; Warfield et al., 2023). Specifically, TAPP (APPSwe-Tau) mice exhibit an association of aggressive behavior with phosphorylated tau localized to lateral parabrachial (LPB) neurons (Warfield et al., 2023). These studies suggest that alterations in various brain regions can contribute to AD-associated aggressive behavior.

### Other NPS-like Behaviors and related pathology in AD mouse models

Other neuropsychiatric symptoms such as sleep disturbances and hyperphagia can be present in AD mouse models. Sleep disturbances are in fact common in many mouse models (Drew et al., 2023; Colby-Milley et al., 2015; Cushing et al., 2020; Holth et al., 2017; Minakawa et al., 2017), for instance, impaired rapid eye movement (REM) sleep has been observed in homozygous APP-NLGF mice (Maezono et al., 2020) and Tg2576 mice (Zhang et al., 2005). Regarding feeding behavior, 3xTg mice consume more food with a rise in metabolic rate as compared to non-transgenic controls (Knight et al., 2012), and the increased feeding could be attributed to defective brain responses to endogenous satiety factors released by food ingestion (Adebakin et al., 2012). In addition, 3xTg mice display decreased ability to sustain their attention, as assessed by a 5-choice serial reaction time test (Romberg et al., 2011). Moreover, behavioral disinhibition has been reported in the APP^SAA^ knock-in model (Xia et al., 2022). Apart from the above NPS-related phenotypes, hallucination- and delusion-like phenotypes have not been thoroughly investigated in AD mouse models due to a lack of reliable behavioral readout.

Pathological examinations have provided neural mechanistic insights into these other neuropsychiatric symptoms. For example, amyloid-β accumulation in the pontine tegmental area and ventral medulla is found to follow a course similar to that of the reduction of REM sleep in APP-NLGF mice (Maezono et al., 2020), while reduced numbers of pedunculopontine tegmentum ChAT-positive neurons correlate with reduced REM sleep in Tg2576 mice (Zhang et al., 2005). The hyperphagia phenotype in female 3xTg mice has been correlated with the number of amyloid-stained-positive cells in the amygdala (Lead Tee and Chiu, 2017). In addition, the disruption of circadian rhythm in 5xFAD mice could be attributed to Aβ-induced degradation of circadian clock regulator molecules such as CBP and BMAL1 (Song et al., 2015). Furthermore, the circadian dysfunction in the Tg2576 model has been associated with compromised GABAergic signaling in the suprachiasmatic nucleus (SCN), a critical structure for orchestrating circadian rhythms (Eeza et al., 2021).

### Synaptic dysfunction underlying NPS in AD

Synaptic dysfunction, a hallmark of AD, plays a crucial role in the NPS observed in various AD models, including 3xTg, APP/PS1, and hAPP J20 models. In the prefrontal cortex of 3xTg mice, elevated spontaneous excitatory postsynaptic currents and firing rates have been observed in pyramidal neurons (Choudhury et al., 2023), and additional studies indicate increased excitatory and inhibitory inputs to layer 2/3 pyramidal neurons, potentially linked to altered social behavior (Bories et al., 2012). In hAPP J20 mice, synaptic transmission and intrinsic excitability are reduced in cortical layer 5 neurons, and specific vulnerability to degeneration is observed in the mPFC (Zhang X.-Q. et al., 2021). These alterations in synaptic activity provide insights into the regional vulnerabilities contributing to NPS in AD. In the hippocampus of multiple models, early and progressive synaptic dysfunctions associated with amyloid and tau pathology have been observed. In the APP/PS1 model, somatostatin (SOM) interneurons near amyloid plaques become hyperactive, while parvalbumin interneurons are hypoactive, leading to an excitation-inhibition imbalance (Algamal et al., 2022). Network disruption also occurs early in 3xTg mice, where GABAergic interneurons in CA1 show reduced inhibition, impacting the overall activation of excitatory neurons (Michaud et al., 2024). In other studies, hippocampal hyperactivity in APP knock-in mice is linked to adenosine deficiency (Bonzanni et al., 2024), while late-stage impairment in long-term potentiation (LTP) is seen in mature APP mice, reflecting deteriorations in synaptic plasticity (Sri et al., 2019). These changes in hippocampal circuitry are mirrored in the amygdala of APOE4 models, where early synaptic deficits further underscore the critical role of synaptic dysfunction in AD progression (Klein et al., 2010). Together, these studies enrich our understanding of how synaptic and network alterations contribute to NPS in AD models.

## Discussion

Neuropsychiatric symptoms are prevalent in AD and cause significant distress to patients and burdens to caregivers. Although they are quite commonly observed in early stages of AD or MCI, the underlying mechanisms are poorly understood. Hence, over the past decade, the NPS has increasingly gained attention in the AD research field.

### Cross-link between NPS and cognitive impairments

A tight interaction between NPS and cognitive decline in AD has been proposed (Siafarikas, 2024). The neuropsychiatric symptoms are commonly associated with accelerated cognitive deterioration (Potter and Steffens, 2007; Lee et al., 2012; Defrancesco et al., 2020), and they could potentially be categorized and associated differentially with AD progression (Potter and Steffens, 2007; Lee et al., 2012; Defrancesco et al., 2020). It has also been shown that the NPS tends to progress in severity and frequency throughout the course of frontotemporal dementia (Benussi et al., 2021). Therefore, NPS may serve as potential biomarkers for predicting the trajectory of cognitive decline in AD or other related dementias (Edwin et al., 2021). Both NPS and cognitive decline are believed to share similar underlying pathological changes, particularly in brain regions such as the prefrontal cortex and hippocampus, which have been implicated in both depression and cognitive function (Campbell and MacQueen, 2004; Trzepacz et al., 2013). Transcranial brain stimulation treatments have been shown to ameliorate both NPS and cognitive symptoms in patients of AD-related dementia (Elder and Taylor, 2014). Additionally, cognitive impairments have also been identified in patients with depression within non-dementia populations (Morimoto et al., 2014), further underscoring the strong correlation between NPS and cognitive dysfunctions. However, more in-depth animal research utilizing techniques such as opto/chemogenetics and *in vivo* imaging is essential to elucidate mechanisms linking NPS to cognitive impairments.

### Comorbidity of NPS features

Studies have shown that NPS features often coexist in AD patients, leading to a complex presentation of symptoms (Edwin et al., 2021; García-Alberca et al., 2011). This increases the complexity of the diagnosis and treatment. Studies have additionally shown that the presence of one NPS feature increases the likelihood of additional symptoms (i.e., anxiety and depression frequently co-occur) (Teri et al., 1999). The comorbidity further suggests that different NPS features may share some common neurobiological pathways. For example, alterations in monoaminergic systems such as serotonin and dopamine may contribute to both anxiety and depression, and neurodegeneration in the prefrontal cortex may lead to both aggression and depression. Moreover, they may share similar genetic risk factors. For example, APOE4 has been identified as a risk factor for both depression (Wang et al., 2019; Zhang J. et al., 2021) and anxiety (Raber, 2007). Future research into these overlapping pathways may help in identifying novel therapeutic targets and strategies for managing NPS in AD more effectively.

### NPS in early-onset and late-onset AD

The profiles of NPS vary between early-onset AD (EOAD) and late-onset AD (LOAD). For example, anxiety is significantly more prevalent in EOAD, with a rate of 70%, compared to 27% in LOAD (Kaiser et al., 2014), and is also more severe in EOAD. EOAD patients generally exhibit higher Neuropsychiatric Inventory (NPI) scores, indicating more severe symptoms, and a higher prevalence of depression, anxiety, apathy, eating problems, and agitation (Baillon et al., 2019; Falgàs et al., 2021; Gumus et al., 2021; Martínez et al., 2021; Polsinelli et al., 2022), although no differences or opposite findings have also been reported (Toyota et al., 2007; van Vliet et al., 2012; Ferreira et al., 2018). These distinctions may be attributed to differences in underlying pathological changes between EOAD and LOAD.

### Promises and difficulties of studying NPS in AD mouse models

Pathological studies in AD patients and AD mouse models have revealed some common changes related to NPS (Table 3). However, investigating NPS-related behaviors in AD mouse models is currently still in its early stages. It should be noted that the currently applied behavioral measurements might not be ideal or even specific for the measurement of any NPS-related feature such as apathy, neither have consistent criteria been used to identify behavioral deficits linked to apathy. As such, phenotypes may not be consistently described for NPS-related deficits across animal models, since some of them might result from motor deficits or other non-cognitive impairments. For example, a decrease in motor activity could reflect less motivation to initiate locomotion, but could also be associated with a deficit in motor function. Regarding the motor activity change of the 5xFAD mouse, both hyper- (Oblak et al., 2021) and hypo- (O’Leary and Brown, 2024) activity has been reported. These apparent discrepancies complicate the interpretation of apathy. Thus, a more robust and specific measurement of apathy or other NPS-related features is urgently needed. The most critical aspect of apathy is the decrease of motivation, and several behavioral assays have been proposed to measure the degree of motivation, for example, the progressive ratio task (Cambre et al., 2023), female encounter test (Ago et al., 2015), latency to enter an arena (Spangenberg and Wichman, 2018), the Switchmaze test (Hartmann et al., 2024), foraging arena test (Xeni et al., 2024) or vision depended behavior (Ortiz et al., 2020). It remains to be determined which one of these is more specific.

**TABLE 3:** Common changes related to NPS between AD patients and AD mouse models.

NPS category	Pathological changes and related brain structures in AD patient	Pathological changes and related brain structures in AD mouse models	Common changes
Apathy	Anterior cingulate cortex, prefrontal regions, putamen, caudate nucleus. Cortical thinning, atrophy, low FA scores, amyloid accumulation, hypometabolism, NFT burden, Dopaminergic dysfunction in basal ganglia, anterior cingulate, and frontal cortices, upregulated 5-HTT sites	High levels of amyloid-beta in prefrontal cortex and hippocampus, dopaminergic neuron degeneration in substantia nigra and VTA	Pathological changes in prefrontal cortex and dopaminergic neuron dysfunction.
Anxiety	Hippocampus, entorhinal cortex, amygdala, prefrontal cortex. Decreased volume, atrophy, hypometabolism. High amyloid-beta accumulation, NFT and A-beta in neocortex, limbic regions, anterior cingulate, entorhinal cortex, serotonergic transporters in the temporal cortex	Neuroinflammation in hippocampus and amygdala, decreased GABA synthesis, amyloid-beta accumulation in hippocampus, altered glutamatergic activity	Pathological changes in hippocampus and amygdala.
Depression	ACC, prefrontal cortex, hippocampus, striatum, amygdala, orbitofrontal cortex. Hypometabolism, structural changes, gray matter atrophy, white matter lesions, altered functional connectivity White matter hyperintensity in occipital area, chronic cerebral hypoperfusion, NFT burden, serotonergic dysfunction, abnormal fatty acids, reduction of antioxidant enzymes	High levels of amyloid-beta in hippocampus and cortex, neuroinflammation, tau pathology, neurotransmitter changes in serotonin and dopamine	Pathological changes in hippocampus and cortex. Serotonergic neuron dysfunction.
Aggression	Amygdala, anterior cingulum, hippocampus, frontal insular, frontal cortex. Atrophy, low fractional anisotropy, increased functional connectivity, amyloid-beta accumulation, hypometabolism. Lowered ChAT activity in frontal and temporal cortex, serotonergic dysfunction, 5-HTT sites preserved, 5-HIAA scores inversely correlated with agitation	Amyloid plaques in nucleus accumbens, cortical, hippocampal and amygdala regions, increased tau pathology, altered neuronal activity in mPFC	Pathological changes in prefrontal cortex, hippocampus and amygdala.
Sleep disturbances	Right middle frontal gyrus, anterior cingulate cortex. Hyperperfusion, hypometabolism. Decreased melatonin in the CSF	Amyloid plaques in hippocampus, GABA-positive astrogliosis in sleep-promoting regions, inflammation in brain and plasma	N/A
Disinhibition	Cingulate, frontal gyri. Atrophy	Alteration of lipids, microglia transcriptomic changes, intracellular amyloid content	N/A

Notably, multiple APP and Tau models exhibit NPS-related deficits, suggesting that these behavioral changes may not be tied to specific pathologies but rather to some shared factors such as alterations in neuronal activity or synaptic functions. Additionally, a single behavioral deficit might indicate changes associated with multiple NPS. For example, a deficit in the sucrose preference test may correlate with both apathy and depression. Although AD animal models hold great promise for elucidating common neural mechanisms, they present significant challenges when investigating NPS, especially in relation to psychosis-related behaviors. One of the main obstacles is the need for more reliable behavioral measurements as readouts. Overcoming these difficulties requires the development of novel behavioral assessments to ensure accurate results.

### Therapeutic potentials based on the understanding of neural mechanisms

As NPS of patients pose a significant source of distress for their caregivers (García-Alberca et al., 2011), the management of NPS would have great beneficial effects to the care provider. Our current understanding of related pathology in AD-NPS points to potential therapeutic targets, such as the monoaminergic system (Delgado, 2000). For example, Rexulti (brexpiprazole) is the first FDA-approved drug for treating AD-associated depression and agitation, and it starts to show some promise (Greig, 2015; Stummer et al., 2020). The drug primarily targets serotonergic and dopaminergic activity, and its co-effectiveness in alleviating depression and aggression further suggests potential shared mechanisms underlying these symptoms.

The excitation-inhibition (E-I) balance is another potential target to consider in the management of AD-NPS. Several lines of evidence suggest that alterations in the E-I ratio of brain circuits could play a major role in neuropsychiatric diseases including depression (Sohal and Rubenstein, 2019; Hu et al., 2023). In AD, an imbalance between excitation and inhibition and hyperexcitability of neural circuits particularly in early stages of the disease (Busche and Konnerth, 2015; Targa Dias Anastacio et al., 2022), caused by the dysfunction of inhibitory neurons (Ambrad Giovannetti and Fuhrmann, 2019), may contribute to neuropsychiatric-like behaviors. Drugs that can affect the E-I balance may alleviate these behaviors. For example, ketamine, via acting on glutamate receptors, can have rapid antidepressant effects (Abdallah et al., 2015). Preliminary investigations suggest that ketamine may provide neuroprotection and reduce neuropsychiatric symptoms associated with AD (Mohammad Shehata et al., 2022).

In conclusion, NPS are prevalent in AD, significantly impacting patients and caregivers. The strong correlation between NPS and cognitive decline underscores the potential of NPS as biomarkers for the trajectory of AD progression. The comorbidity of NPS features suggest shared neurobiological pathways, while their varied presentations in early-onset and late-onset AD suggest different genetic risk factors. Although animal models present challenges in studying NPS, they offer invaluable insights into the potential underlying mechanisms. Advancements in understanding these mechanisms highlight therapeutic potentials of targeting the monoaminergic systems and the E-I balance. Continued research along these directions is essential to develop effective treatments for managing NPS in AD.

## Author contribuitons

NZ: Writing – original draft, Writing – review and editing, Conceptualization, Data curation, Formal Analysis, Investigation, Methodology, Resources, Software, Validation, Visualization. SZ: Writing – original draft, Writing – review and editing, Conceptualization, Data curation, Formal Analysis, Investigation, Methodology, Resources, Software, Validation, Visualization. LZ: Writing – original draft, Writing – review and editing, Conceptualization, Data curation, Formal Analysis, Funding acquisition, Investigation, Methodology, Project administration, Resources, Software, Supervision, Validation, Visualization. HT: Conceptualization, Data curation, Formal Analysis, Funding acquisition, Investigation, Methodology, Project administration, Resources, Software, Supervision, Validation, Visualization, Writing – original draft, Writing – review and editing. G-WZ: Conceptualization, Data curation, Formal Analysis, Funding acquisition, Investigation, Methodology, Project administration, Resources, Software, Supervision, Validation, Visualization, Writing – original draft, Writing – review and editing.
